# Molecular docking and GC-MS data for the inhibition of RAD51 expression by a compound from Clerodendrum inerme L.

**DOI:** 10.6026/97320630017767

**Published:** 2021-08-31

**Authors:** Karthikeyan Kalimuthu Ayyakkalai Marikkannu, Sasireka Ganesan

**Affiliations:** 1 Department of Zoology, N.M.S.S.Vellaichamy Nadar College, Madurai-625019, Tamil Nadu, India; 2 Department of Zoology, Sri Meenakshi Government Arts College for Women, Madurai-625002, Tamil Nadu, India

**Keywords:** C. inerme extract, GC-MS analysis, Molecular docking, ADME properties

## Abstract

Screening of potential inhibitors for RAD51 from petroleum ether extract of Clerodendrum inerme L. (C.inerme) is of interest. Presence of phytocompounds was identified using GC-MS analysis. Molecular docking and ADME properties were calculated for potential inhibitors for RAD51. A total of 25 phytocompounds were extracted from the petroleum ether extract of C.inerme. The compound 1,2,4-Trimethyl-3-nitrobicyclo [3.3.1]nonan-9-one shows binding features with the cancer target protein RAD51 similar to the FDA approved drug of 5-Flurouracil for further consideration in the context of pancreatic cancer drug discovery.

## Background:

Cancer is a leading cause of death worldwide and projected to continue rising with an estimate of 12 million deaths in 2030 [1]. Pancreatic cancer is the fourth leading cause of cancer deaths with one of the worst survival rates of all cancer types [2]. According to World Health Organization, it is estimated that around 330 372 [3]. In 2020, the American Cancer Society estimated that around 4,16,420 people will be diagnosed with pancreatic cancer and approximately 39,590 people will die of pancreatic cancer. Pancreatic adenocarcinoma has the highest mortality rate of all human cancers [4]. Overexpression of RAD51 occurs in variety of cancers, which includes pancreatic cancer and is a negative prognostic marker for the survival of various cancer patients. Increased expression of RAD51 and other HRR-associated genes in tumor cells is supposed to enhance DNA repair and increase resistance to DNA damaging substances [5]. Currently, there is no efficient drug to treat against pancreatic cancer. Since ancient times, people have been exploring the nature particularly medicinal plants in search of new drugs [6]. However, Natural Products traditionally have played an important role in drug discovery. Plants have been utilized as medicines for thousands of years and these medicines initially took the form of crude drugs such as tinctures, teas, poultices, powders and other herbal formulations [7]. Natural products are often obtained by conventional approaches involving extraction and separation techniques, such as the use of organic solvents to extract the material [8]. Herbal medicine is still the mainstay of about 75–80% of the world population, mainly in the developing countries, for primary health care because of better cultural acceptability, better compatibility with the human body and lesser side effects [9]. Thus, it leads to sudden increase in the number of herbal drug manufactures. Hence, there is need of effective anti-pancreatic drug from natural compounds to treat this fatal disease. Clerodendrum inerme (C.inerme) is the medicinal plant, which belongs to the family of Verbenaceae. It is commonly known as Wild Jasmine grows in the Indian subcontinent and around the world (Australia, Asia, Malaysia, and the Pacific islands) [10]. Different parts of this plant are used in the treatment of rheumatism, skin disease, venereal infections, beriberi, and tumors [11]. Crude extract of C.inerme possess antioxidant, antimicrobial, anticancer and immunomodulatory activity [12]. Therefore, it is of interest to document the molecular docking and GC-MS data for the inhibition of RAD51 expression by 1,2,4-Trimethyl-3-nitrobicyclo[3.3.1] nonan-9-one from C. inerme.

## Materials & Methods:

### Plant collection:

The aerial parts of C. inerme was procured from in and around the Poonthottam, Thanjavur District, Tamil Nadu, India. The whole plant material was washed thoroughly under running tap water, air-dried, finely powdered and stored in airtight bottle for further studies.

### Preparation of petroleum ether extract:

The plant powder was soaked in n-hexane solvent and kept in the shaker for 48 hr at room temperature. The extract was collected and concentrated at 40°C under reduced pressure using rotary evaporator. The dried extract was stored at 4°C until further use. The remaining residue was extracted 3 times with the fresh solvent to ensure complete extraction.

### GC-MS analysis:

GC-MS analysis of n-hexane extract of C.inerme was performed using the equipment Agilent technologies 7890 A. The equipment has a DB 35- MS Capillary Standard non-polar column with dimensions of 30 mm x 0.25 mm IDx0.25 µm film. The carrier gas used is Helium with at low of 1.0 ml/min. The injector was operated at 250°C and the oven temperature was programmed as follows: 60°C for 15 min, then gradually increased to 280°C at 3 min. The identification of components was based on Willey and NIST libraries as well as comparison of their retention indices. The constituents were identified after comparison with those available in the computer library (NIST and Willey) attached to the GC-MS instrument and the results obtained have been tabulated [13].

### Preparation of protein structure:

The 3D structure of RAD 51 (Protein Data Bank Identifier) PDB ID: 1NOW was retrieved from the Protein Data Bank and the protein was prepared by protein preparation wizards (standard methods) that are available in grid-based ligand docking with energetics (Protein preparation wizard, Schrodinger, 2018). Protein was optimized using sample water orientation and minimized by using (Root-Mean Square Deviation) RMSD 0.30 Å and (Optimized Potentials for Liquid Simulations) OPLS (2005) force field.

### Active site prediction:

The active site (binding pocket) and functional residues of RAD 51was identified and characterized by site-map module from Schrodinger package (Schrodinger, 2012). SiteMap calculation begins with an initial search step that identifies or characterizes- through the use of grid points- one or more regions on the protein surface that may be suitable for binding ligands to the receptor. Contour maps were then generated, produced hydrophobic, hydrophilic maps hydrogen binding possibilities, which may guide the protein-ligand docking analysis.

### Ligand preparation:

The active 25 phytocompounds were identified from extract of C.inerme by GC-MS analysis. The screened compounds were used in molecular docking studies. The phytocompounds and (Food and Drug Administration) FDA approved drug i.e., 5-flurouracil were used in molecular docking studies. These ligands were prepared using the LigPrep 2.4. The structure of each ligand was optimized by means of the OPLS 2005 forcefield using a default setting.

### Molecular docking analysis:

All docking analysis was performed by using the Standard Precision (SP), which is Standard mode of Glide 5.6 (Grid-based Ligand Docking with Energetic) module from Schrodinger package. The phytocompounds and FDA approved drug were docked into the binding site of RAD 51 using Glide 5.6. The scaling Vander Waals radii were 1.0 in the receptor grid generation. Grid was prepared with the bounding box set on 20Å. The co-ordinates of this enclosing box with the help of the active site residues to be set default. The force field OPLS_2005 was used for docking protocol and the lowest-energy docked complexes were found in the majority of similar docking conformations.

### ADME properties prediction:

The selected phytocompounds and FDA approved drug were checked for their ADME properties using QikProp 2.3 module. QikProp helps in analyzing the pharmacokinetics and pharmacodynamics of the ligand by accessing the drug like properties. Predicted significant ADME properties such as Molecular weight (MW), H-Bond donor, H-Bond acceptor and log P (O/W).

## Results and Discussion:

GC-MS analysis showed that the petroleum ether extract of C.inerme contains around 25 phytocompounds which are 2-Hexadecen-1-ol, 3,7,11,15-tetramethyl-, [R-[R*,R*-(E)]]- (15.89 %), 3-exo-(Aminomethyl)tetracyclo-[6.3.1.0(2,6).0(5,10)]dodecan-3-en do-ol (12.42 %), (-)-Caryophyllene oxide (11.64 %), (+)-2-endo,3-endo-dimethylbornane (10.93 %), 4-(4-Chlorobenzoyl)-1-cyclohexyl-5-tosylamino-1H-1,2,3-triazole (8.00 %), (E)-13-methyl-11-tetradecen-1-ol acetate (6.04 %), Exobornyl acetate (3.93 %), Hexadecanoic acid, methyl ester (3.67 %), 2-Hexadecen-1-ol, 3,7,11,15-tetramethyl-, [R-[R*,R*-(E)]]- (3.12 %), (E)-4-(2',2',6'-TRIMETHYL-5'-OXO-1',6'-EPOXY-1'-CYCLOHEXYL)-3-BUTEN-2-ONE (2.71 %), Dodecanoic acid, methyl ester (2.55 %), aromadendrene 2 (2.34 %), 2(4H)-Benzofuranone, 5,6,7,7a-tetrahydro-4,4,7a-trimethyl- (1.97 %), Dimenthyl acetylenedicarboxylate (1.77 %), 3-Cyclohexene-1-methanol,4-trimethyl-, (S)- (1.66 %), 4-exo-4-Cyano-2-aza-3-oxatetracyclo[7.3.1.1(7,11).0(2,6)]tetradecane (1.32 %), Decane (1.28 %), 5,5"-Diethynyl-2,2':6',2"-terpyridine (1.21 %), 4-Oxanaphtho[2,1-c]pyridine-3-one (1.19 %), Butanamide, 4-cyano- (1.14 %), Benzene, 1-(1,5-dimethyl-4-hexenyl)-4-methyl- (1.14 %), (trans)-3-[Trifluoromethoxycarbonyl]-5-(methoxycarbonyl)-1-cyclohexene (1.06 %), rac-1,2,4-Trimethyl-3-nitrobicyclo[3.3.1]nonan-9-one (1.05 %), 5,5''-Dichloro-4'-hexoxymethyl-2,2':6,2"-terpyridine (0.99 %), 1,3-Bis(diethylphosphono) difluoromethyl]benzene (0.97 %) and all the compounds were analysed for anti-pancreatic cancer activity by the molecular docking analysis. Based on glide score and glide energy, the docking results were analyzed.

**Figure 1:**
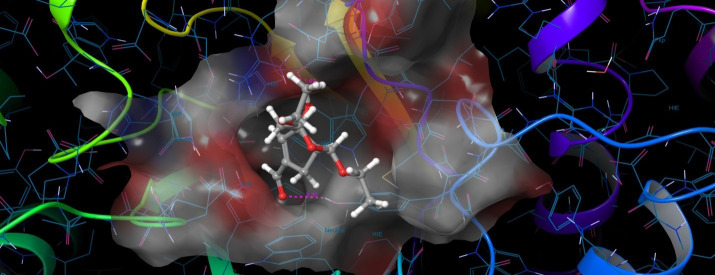
The 3D structure of 1,2,4-Trimethyl-3-nitrobicyclo[3.3.1]nonan-9-one/RAD51 complex.

**Figure 2:**
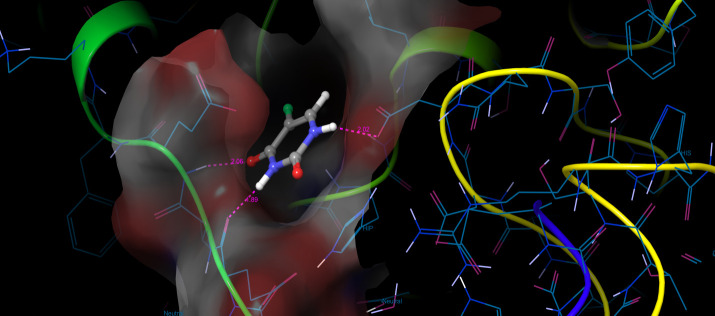
The 3D structure of 5-flurouracil/RAD51 complexes.

The molecular docking results revealed that RAD 51 (Figure 1) complexed with 1,2,4-Trimethyl-3-nitrobicyclo [3.3.1]nonan-9-one has good glide score of -6.16 Kcal/mol and the glide energy was -32,89 Kcal/mol when compared with FDA approved drug ie., 5-flurouracil complex with RAD 51 (Figure 2) has low glide score and glide energy of -5.87 / -28.47 Kcal/mol respectively. In RAD51/1,2,4-Trimethyl-3-nitrobicyclo[3.3.1]nonan-9-one complex TYR450 and GLU491 residues strongly bind with C=O and OH respectively. Moreover RAD51/5-flurouracil complex GLU357 residue makes a hydrogen bond with C=O and ASP241 and GLU355 residues bind with NH on the complex. Pancreatic cancer is a major cause of cancer related mortalities accounting for approximately 6% of all cancer-related deaths in men and women [14]. Due to late-stage diagnosis most patients have limited chance for curative resection, thus novel approaches for early detection and effective treatment must be explored [15]. Traditional plant-based medicines still put forth a great deal of importance to people living in developing countries leading to the discovery of new drug candidates [16]. Various studies reported that these computational techniques could strongly support and help the design of novel, more potent inhibitors by revealing the mechanism of drug-receptor interaction [17]. Computational biology and bioinformatics have not only the potential to speed up the drug discovery process thus reduces the cost but it also facilitates and speed up the drug designing process [18]. However, recent advancements in synthesis of compounds and large numbers of new compound libraries currently available for biological screening, poses a high demand for predictive computational methods that can prioritize molecules for biological screening [19].

RAD51 is a potential therapeutic target, which is overexpressed in pancreatic cancer. Recent studies suggested that RAD51 is overexpressed and contributes to the development, progression and drug resistance of pancreatic cancer cells. The role of RAD51 in pancreatic tumorigenesis and drug resistance, and hypothesize that RAD51 could serve as a potential biomarker for diagnosis of pancreatic cancer. Furthermore, RAD51 protein, a key player in the HR mediated. DSB (double strand breaks) repair has been found to be overexpressed in pancreatic cancer [20]. Screened phytocompound inhibits RAD51 with comparable docking score and docking energy; hence it might act as potential inhibitor for RAD51. Absorption, distribution, metabolism, excretion and toxicity (ADME) properties should be considered to develop a new drug, because they are the main cause of failures for candidate molecules in drug design [21]. The natural compound of 1,2,4-Trimethyl-3-nitrobicyclo [3.3.1] nonan-9-one was within the acceptable range (results shown in Table 2 - see PDF) The limitations of ADME properties are: not more than 5 hydrogen bond donors; not more than 10 hydrogen bond acceptor; molecular mass less than 500 daltons; an octanol- water partition coefficient logP not greater than 5.

## Conclusion:

We document the molecular docking and GC-MS data for the inhibition of RAD51 expression by 1,2,4-Trimethyl-3-nitrobicyclo [3.3.1] nonan-9-one from C. inerme in the context of pancreatic cancer drug discovery.
